# Frequency and Outcomes of Acute Kidney Injury in the First Month Post-transplant: A Study on Renal Transplant Recipients in a Resource-Limited Country

**DOI:** 10.7759/cureus.80277

**Published:** 2025-03-09

**Authors:** Zoha Zafar, Adil Manzoor, Rabia Shahid

**Affiliations:** 1 Nephrology, Pakistan Kidney and Liver Institute and Research Centre, Lahore, PAK

**Keywords:** acute kidney injury, cni toxicity, kidney transplant, post-renal transplant, serum creatinine, urinary tract infection

## Abstract

Objective

The study aimed to determine the incidence of acute kidney injury (AKI) in renal transplant recipients in the first month after transplant. The number of AKI episodes per patient and their outcome on renal graft function were also determined.

Material and methods

It is a retrospective study that took place in the Nephrology Department of Pakistan Kidney and Liver Institute and Research Center (PKLI & RC), Lahore, Pakistan. A total of 195 patients aged 18-70 years who underwent kidney transplant surgery at PKLI & RC, Lahore, were selected in this cohort that underwent renal transplants from 31st January 2024 to 31st December 2024. One month post-transplant course was followed by obtaining serum creatinine level values. Data was analyzed using Microsoft Excel (Microsoft® Corp., Redmond, WA, USA).

Results

A total of 81 out of 195 patients (41.5%) had AKI within the first 30 days following a renal transplant. Seventy patients experienced AKI once (86.4%), meanwhile, 11 patients (13.5%) had two episodes of AKI within the first 30 days. Staging done as per Kidney Disease Improving Global Outcomes (KDIGO) guidelines showed that 73 patients had stage I AKI (90.1%). Three patients had stage II AKI (3.7%), while five patients had stage III AKI (6.2%). The most common cause was found to be pre-renal (dehydration) in 24 patients out of 81 (29.6%) and followed by a urinary tract infection in 23 patients (28.3%). Twenty patients (24.6%) had drug-induced AKI; there was calcineurin inhibitor (CNI) toxicity in 8.6% and acute tubular necrosis (ATN) in 7.4% of patients. One patient had acute antibody-mediated rejection (ABMR). Most cases of AKI were found to be self-limiting, with complete resolution to baseline renal allograft function.

Conclusion

Even though most episodes of AKI completely resolved to baseline creatinine, it is pivotal to timely diagnose and treat AKI in post-renal transplant patients. If left untreated, there can be a worsening of graft function and overall outcome of the transplant.

## Introduction

The global estimated prevalence of chronic kidney disease (CKD) is 13.4% (11.7-15.1%) [1], with the majority of the disease burden prevalent in low- to middle-income countries [2]. Lack of proper follow-up with the physician and the rampant pseudo-sciences undoubtedly add fuel to the fire. Renal replacement therapy, comprising of dialysis (hemodialysis and peritoneal dialysis) and renal transplant, is the mode of treatment in end-stage renal disease (ESRD) [3]. While a renal transplant is the ultimate life-saver in such a scenario, one cannot deny the rocky road that might await one after transplantation. From subclinical rejection to full blown graft failure, there is a whole universe of post-renal transplant complications that a patient and physician might encounter [4]. Amidst this healthcare crisis, the advent of modern-day transplant nephrology has helped to tame this beast of complicated transplants. Allograft and patient survival has improved by leaps and bounds from its nascent days due to an increased knowledge of immunology and infectious diseases. These two aspects are notorious for complicating the post-transplant course of a renal transplant recipient [5]. One of the most common complications encountered is acute kidney injury (AKI) [6,7] - an acute insult that hampers kidney function - and may deteriorate the graft health in the longer run. Studies done globally have shown that 40-85% of renal transplant recipients experience at least one episode of AKI in their lifetime [8]. As per Kidney Disease Improving Global Outcomes (KDIGO), AKI is defined as a sudden decrease in kidney function. This can be identified by an increase in the value of serum creatinine or a decrease in the patient's urine output. Urine output measurement is not always possible and/or reliable in outpatient settings, so measurement and comparison of serum creatinine value is the method used widely to identify an episode of AKI. Furthermore, AKI is staged as per severity of insult.

Stage I is characterized by an increase in serum creatinine value by more than 0.3 mg/dL, or an increase in value from 1.5 to 1.9 times of baseline creatinine. Stage II AKI is labelled when a patient's blood work shows a 2.0 to 2.9 times increase in serum creatinine from baseline. Stage III AKI refers to a three times increase in serum creatinine value or an absolute value of >4mg/dL or initiation of renal replacement therapy [9].

A sudden decline in urine output can also be used to stage AKI. However, for the purpose of this study, AKI diagnosis is made by following serum creatinine value of the patient over time. There is a scarcity of local data on this subject. We aim to look for incidences of AKI in post-transplant recipients in the first month after transplant surgery and the outcome of each episode.

The causes of AKI specific to the renal transplant include ischemia-reperfusion injury, acute rejection, acute calcineurin inhibitor (CNI) toxicity, venous or arterial thrombosis, urinary tract obstruction, urinary tract infection (UTI), graft pyelonephritis, and recurrent disease. As in the general population, post-transplant AKI is associated with a higher incidence of CKD. Renal transplant recipients with AKI are at increased risk of graft loss and death.

## Materials and methods

This is a retrospective cohort study done on 195 patients who underwent kidney transplant surgery at the Pakistan Kidney and Liver Institute and Research Centre (PKLI & RC) in Lahore, Pakistan, from January 2024 to December 2024. We followed the one-month post-transplant course of the patients that fulfilled our inclusion criteria. The incidence of AKI, number of episodes per patient, and the outcome on allograft function after AKI are determined in this study. The definition and staging of AKI are done as per KDIGO guidelines. Only serum creatinine value is used for the purpose of this study, and urine output is not taken into account due to inconsistencies with the quantification.

All patients’ serum creatinine level was checked immediately following renal transplant, and repeat serum creatinine values at 7th, 14th, 21st, 28th, and 30th post-transplant day were followed for determining the incidence of AKI among post-transplant patients. The number of episodes per patient was also documented, and the subsequent outcome after each episode was followed.

Inclusion criteria

Patients aged 18-70 who had kidney transplant surgery at PKLI & RC were included in this study.

Exclusion criteria

Renal transplant patients below 18 years of age at the time of transplant, those admitted for close monitoring immediately after renal transplantation, patients with known irreversible graft failure within the first month of renal transplant, and those who underwent a combined liver and kidney transplant were excluded.

Statistical analysis

Descriptive statistics was used to summarize the findings. The incidence of AKI and the number of episodes per patient have been calculated using Microsoft Excel (Microsoft® Corp., Redmond, WA, USA). Stratification of severity was done on the basis of the stage of AKI as defined in KDIGO guidelines. The various contributing and causative factors are presented in the form of percentages.

## Results

The study consisted of 195 patients who underwent a renal transplant at PKLI & RC, Lahore, and fulfilled the inclusion criteria. Demographic analysis showed that 84% of the patients were male (n=164) and 26% were female (n=31). The mean age of the patients was found to be 34.4 years. Twenty-five patients had undergone preemptive transplant (12.8%), while the remaining 170 were dialysis-dependent (87.1%) before transplant. Duration on dialysis varied from two months to six years, with the average duration on dialysis being 23 months.

Incidence and frequency of AKI

Mean baseline creatinine was 1.1 mg/dL, which was achieved between days 3 and 7 following the renal transplant. AKI occurred in 41.5% (n=81) of patients within the first 30 days following a renal transplant. The number of patients who experienced AKI once during the first 30 days were 70 (86.4%). While 13.5% of patients (n=11) had two episodes of AKI in the same duration.

Stage of AKI

**Figure 1:**
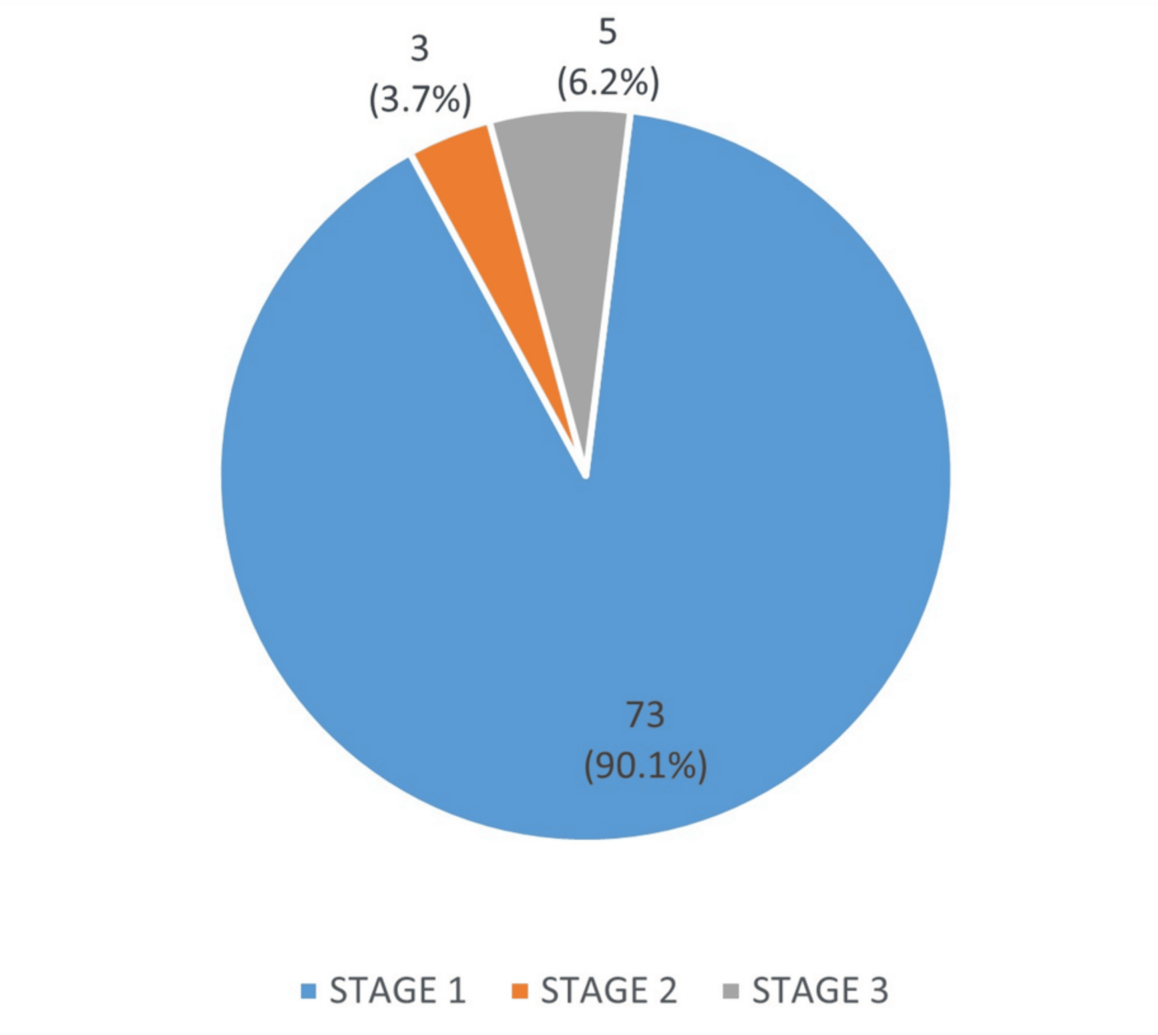
Stages of Acute Kidney Injury in Post-renal Transplant Recipients

The staging of AKI as per KDIGO guidelines showed that 73 patients had stage 1 AKI (90.1%). Three patients had stage 2 AKI (3.7%), while five patients were found to have stage 3 AKI (6.2%), as per KDIGO guidelines. Only one patient required a dialysis session after a renal transplant due to delayed graft function. As illustrated in Figure 1, the distribution showed that most cases of AKI were limited to stage 1.

Causes of AKI

**Figure 2:**
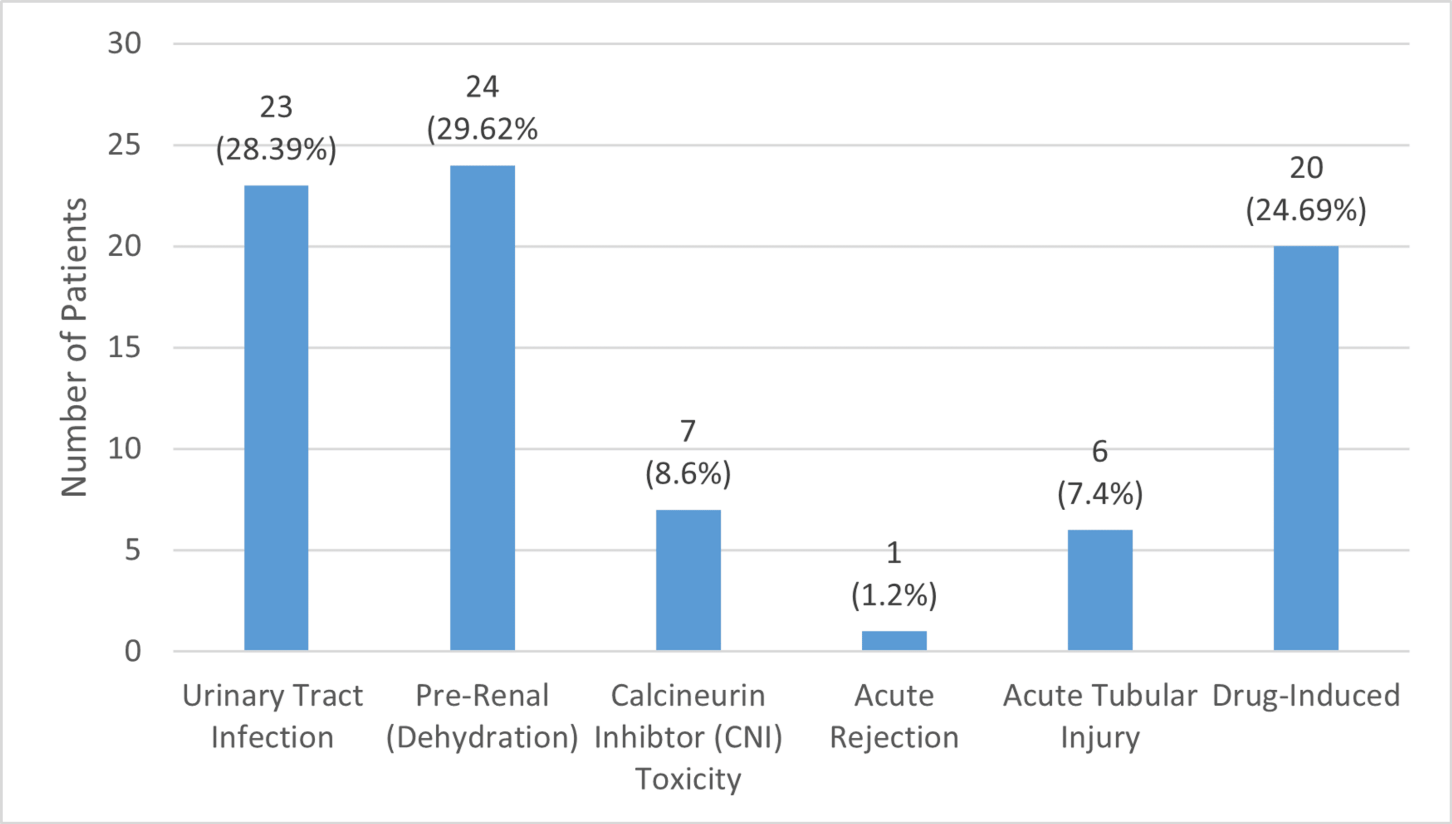
Causes of Acute Kidney Injury in Post-renal Transplant Recipients

There were various causes of AKI in renal transplant recipients. The most common cause of AKI was pre-renal (dehydration) in 24 patients out of 81 (29.62%). The second most common cause was found be a UTI/urosepsis in 23 patients (28.39%). There was drug-induced AKI in 20 patients (24.69%), where the most notorious was found to be co-trimoxazole, which had been given prophylactically. CNI toxicity in 8.6% (n=7) of patients caused AKI, followed by acute tubular injury/necrosis (ATN) diagnosed on renal biopsy in 7.4% (n=6) of patients. Only one patient had an episode of acute antibody-mediated rejection (ABMR) that caused AKI. Rejection was diagnosed on biopsy and correlated with the high mean fluorescence intensity (MFI) of donor specific antibodies (DSA) in the patient's blood after transplant. Figure 2 represents this data, showing the distribution of causative factors of AKI in post-renal transplant patients.

## Discussion

The incidence of AKI within the first 30 days after renal transplant was found to be 41.5%. While the majority of these episodes were mild to moderate, consisting of stage 1 AKI (90.1%), it is critical to note that they resolved after due changes were made to their care plan moving forward. Similar findings have been reported in international studies [10]. Each patient scenario is unique, and no general practice can be applied for prevention of AKI in post-transplant course.

The anatomy and physiology of the nephron are woven so intricately that the proper functioning of every part is critical to its optimum functioning. This also leaves it susceptible to injury at every point. When the allograft is donated from the donor (living donor in this particular study), the cold ischemia time starts. The variability in cold ischemia time can result in significant post-operative complications and a delayed graft function [11]. Once the surgical anastomosis has been secured, there is a risk of ischemic reperfusion injury [12]. Tubules of the nephron are particularly vulnerable to insult.

Acute rejection was one of the main causes of AKI and declining graft function in renal transplants in the past [13,14]. With advancing knowledge of transplant immunology, we have curbed the disease burden posed by immediate post-transplant acute rejection. Now there is a vigilant pre-transplant evaluation process which aids in selecting the best recipient - donor match by taking blood group (ABO) compatibility and minimal immunological risk into consideration. Human leukocyte antigens (HLA) matching is done along with Luminex screening and flow cytometry (cross-match) before proceeding with the transplant. This practice has helped to reduce the risk of rejection to a negligible amount. ABMR is a type of rejection where the host immune system starts making antibodies against the antigens present in the donor kidney [15]. If there are DSA in the patient's serum before transplant, the MFI predicts the type of desensitization protocol that should be implemented. Desensitization protocols are implemented in patients undergoing higher immunological risk transplants [16]. Some cases proceed without desensitization, and a higher dose of induction therapy (anti-thymocyte globulin in this study) is put into the plan. Induction therapy and the future immunosuppression target are based on the recipient-donor pair tissue typing.

In the post-transplant follow-up, a personalized prescription is made and readily modified for each recipient based on their immunological response and tissue matching. Younger patients' robust immune system demands a higher level of immunosuppression. Tacrolimus is a CNI used for maintenance immunosuppressant therapy in renal transplantation. Its level is monitored by the blood concentration of the drug (trough level), which is estimated right before the next dose is to be taken (approximately 12 hours after the last dose). There is a wide range of inter- and intra-patient variability with this drug [17]. Transplant nephrologist has to employ a trial-and-error management to achieve the desired levels of tacrolimus. Few patients suffered an acute insult after getting persistent supra-therapeutic tacrolimus levels and resulting CNI toxicity. Sometimes, there has to be a shift in immunosuppressive therapy as best suited for the kidney function, and CNI has to be changed if repeated insults are noted to the graft [18]. Mammalian target of rapamycin (mTOR) inhibitors such as sirolimus are another option in such a scenario. The drug works by inhibiting the function of mTOR, which is a protein kinase that helps in the growth and survival of cells.

Pre-renal causes of AKI include dehydration [19], which can be caused by multiple factors. Few immunosuppressive drugs, such as mycophenolate mofetil, are notorious for their undesirable gastrointestinal symptoms, including profuse watery diarrhea [20]. *Cytomegalovirus* (CMV) and *Clostridium difficile* (toxins A and B) can also cause diarrhea in a post-renal transplant patient [21]. Pre-transplant risk stratification for CMV is done by checking serum IgG and IgM levels. Post-transplant prophylaxis with low-dose valgancyclovir is given according to the particular risk status pertaining to CMV in the patient. Reduction in mycophenolate mofetil or changing the salt to enteric-coated formulation mycophenolic acid sodium sometimes proved beneficial in limiting the GI symptoms faced by the patient. Timely oral and/or intravenous hydration was helpful in the majority of patients who suffered from pre-renal AKI [19].

Co-trimoxazole is another prophylactic antibiotic given to renal transplant recipients to ward off potential infections in an immune-suppressed individual. However, the drug has known side effects, including hemodynamic instability and acute tubular injury. The drug is renowned for its hyperkalemic effect [22]. Its inhibiting role in creatinine secretion can cause a resultant rise in serum creatinine level and paint the picture of cute AKI even in the absence of a true decline in the glomerular filtration rate (GFR) of the allograft [23]. It was observed that even slight modifications to the regime, such as holding co-trimoxazole for a few days, resolved the picture of AKI, and serum creatinine level returned to baseline. In the interim, no other prophylaxis was started in place of co-trimoxazole. The drug was resumed after the serum creatinine down-trended, sometimes in thrice weekly frequency to balance benefits with potential side effects. It can not be said with certainty whether it was a true AKI or just a rise in creatinine, due to lack of resources where renal biopsy can not be performed for every case with a slight increase in serum creatinine.

Infection can trigger an immune response and ultimately pose a threat to the allograft by causing AKI. Urinary tract infections (UTI) are more common in the immediate post-transplant phase. Their incidence can vary from 26% to 79% in the first year and subsequently decrease with each passing year [24]. An infected double J stent (DJ stent) was found responsible in many cases, where source control with removal of stent and antibiotic therapy resolved the infection/sepsis. The most common pathogens identified were gram-negative bacteria such as *Escherichia coli* (*E. coli*), followed by *Klebsiella pneumoniae* (KP), *Proteus*, and *Pseudomonas aeruginosa*. *Enterococcus* species were also frequently present in the culture of such patients, with some having vancomycin-resistant *Enterococcus* (VRE) as a catheter-associated UTI (CAUTI). Efficient infection prevention and control methodologies, such as the application of different sepsis care bundles, were applied during hospital stays to minimalize the incidence of hospital-acquired infections.

A critical aspect of transplant nephrology is to keep a hawk’s eye on the patient’s post-operative signs and symptoms, combined with the laboratory investigations. Declining serum creatinine is not the only marker of AKI. A few cases might be undergoing a tissue-level insult, which can only be diagnosed with histopathology after renal biopsy [25]. However, following the trend of serum creatinine is the best approach in a resource-limited setting where renal biopsy is not readily available and affordability issues come into play. A properly optimized pre-transplant evaluation plays a pivotal role in identifying potential threats to post-transplant allograft function.

There are potential limitations in this study that we identified. As per the conventional definition of AKI, the increase of 0.3 mg/dL from baseline is a cut-off value, however, it is a debatable aspect in post-renal transplant patients. Considering that the baseline creatinine is, in reality, a variable range rather than one figure, there is potential for misdiagnosing a normal variation as AKI. Some patients reach baseline creatinine later than expected, and the seemingly variable value can be misinterpreted as AKI. Laboratory variations owing to different calibrations, instruments, and processing delays can also hamper the authenticity of serum creatinine value of a patient.

An important aspect that was missed in this study and could not be accounted for is the urine output of patients. KDIGO guidelines also describe the decrease in urine output as a measure of AKI staging. However, this estimation could not be done due to patients not being compliant with urine output monitoring once they were discharged from the hospital after a transplant. Even with patients' compliance, there is a margin of error in the results, and they are not reliable enough to be used for conclusive decision-making.

Renal biopsy can aid in reaching a definitive diagnosis of AKI in some patients, as sub-clinical tissue damage can only be identified via histopathology. There is a limitation to the number of renal biopsies being done in third-world countries due to financial constraints and affordability dilemmas. More often than not, it is upon the clinical judgment of the treating physician to go ahead with a few biopsies that are expected to yield fruitful results and important information regarding the allograft function. It is imperative that some patients with minimal variation in serum creatinine are overlooked, where there might be potential tubular injury on a microscopic level.

There is a gap in the existing data regarding the incidence of AKI in renal transplant recipients in low- to middle-income countries due to the limited number of transplants being done. Future studies should be conducted with a bigger cohort to generalize the findings and identify areas of potential threat to the allograft health.

## Conclusions

In order to reduce the burden of disease and reduce the post-transplant complications, it is critical to diagnose and treat AKI. If left untreated, there can be worsening graft function and overall outcome of renal transplant. The study showed a staggering 41.5% incidence of AKI in renal transplant recipients during the first month after their surgery.

Most patients only had one episode of AKI during this duration (86.4%), while 13.5% of patients had two episodes of AKI within the first 30 days. The most frequently encountered cause was pre-renal due to dehydration, followed by UTI in 28.4% of patients. The start of an immunosuppressive regime has its own set of known side effects, including GI disturbances and tubular injury. It is crucial to keep a close follow-up in the early post-operative duration to limit such episodes and prolong the life of the graft.
